# Non-cancer-related pathogenic germline variants and expression consequences in ten-thousand cancer genomes

**DOI:** 10.1186/s13073-021-00964-1

**Published:** 2021-09-09

**Authors:** Zishan Wang, Xiao Fan, Yufeng Shen, Meghana S Pagadala, Rebecca Signer, Kamil J. Cygan, William G. Fairbrother, Hannah Carter, Wendy K. Chung, Kuan-lin Huang

**Affiliations:** 1 Department of Genetics and Genomic Sciences, Center for Transformative Disease Modeling, Tisch Cancer Institute, Icahn Institute for Data Science and Genomic Technology, Icahn School of Medicine at Mount Sinai, New York, NY 10029 USA; 2 Departments of Pediatrics and Medicine, Columbia University Irving Medical Center, New York, NY 10032 USA; 3 Departments of Systems Biology and DBMI, Columbia University Irving Medical Center, New York, NY 10032 USA; 4 Department of Medicine, University of California San Diego, 9500 Gilman, San Diego, CA 92093 USA; 5 Department of Molecular Biology, Cell Biology and Biochemistry, Brown University, Providence, RI USA

## Abstract

**Background:**

DNA sequencing is increasingly incorporated into the routine care of cancer patients, many of whom also carry inherited, moderate/high-penetrance variants associated with other diseases. Yet, the prevalence and consequence of such variants remain unclear.

**Methods:**

We analyzed the germline genomes of 10,389 adult cancer cases in the TCGA cohort, identifying pathogenic/likely pathogenic variants in autosomal-dominant genes, autosomal-recessive genes, and 59 medically actionable genes curated by the American College of Molecular Genetics (i.e., the ACMG 59 genes). We also analyzed variant- and gene-level expression consequences in carriers.

**Results:**

The affected genes exhibited varying pan-ancestry and population-specific patterns, and overall, the European population showed the highest frequency of pathogenic/likely pathogenic variants. We further identified genes showing expression consequence supporting variant functionality, including altered gene expression, allelic specific expression, and mis-splicing determined by a massively parallel splicing assay.

**Conclusions:**

Our results demonstrate that expression-altering variants are found in a substantial fraction of cases and illustrate the yield of genomic risk assessments for a wide range of diseases across diverse populations.

**Supplementary Information:**

The online version contains supplementary material available at 10.1186/s13073-021-00964-1.

## Background

Recent advances in sequencing technology and targeted therapeutic development have led to increased clinical genomic sequencing for patients. In 2015, the American College of Molecular Genetics and Association for Molecular Pathology (ACMG/AMP) established criteria for genetic variant classification on a spectrum from pathogenic to benign as a guide for consistent clinical interpretation [1]. The ACMG also curated a list of 59 medically actionable, highly penetrant genes (abbreviated ACMG 59 genes) [2], for which reporting of secondary findings is recommended. However, pathogenic variants in non-cancer or cancer-syndrome-related genes have rarely been systematically evaluated in a large cohort of cancer patients across diverse ancestries. Furthermore, many variants are extremely rare, and might be founder variants exclusive to a specific ancestral population [3]. Extensive genetic analyses across diverse populations will help to inform future population-based genomic screening strategies.

Beyond identifying DNA sequence variant, variant interpretation often requires assumptions about the expression consequences of the variants. For example, many pathogenic variants are premature stop-codons presumed to cause mRNA transcript degradation through nonsense-mediated decay (NMD) [4]. The diminished gene expression impairs cellular function, leading to disease phenotype. Concurrent observation of expression aberrations in tissue samples—at both the allelic specific expression (ASE) [5, 6] and gene expression level—can validate variant functionality. Yet, published large-scale studies that evaluate expression consequences of genomic variants mostly represent European populations with available multi-omics data like Genotype-Tissue Expression (GTEx) project [7–10]. The Cancer Genome Atlas (TCGA) project includes DNA/RNA-Seq data for over 10 K cancer cases of multiple ancestries [11], providing an opportunity to dissect the expression consequences of variants.

In this study, we systematically investigated pathogenic or likely pathogenic genetic variants in human disease genes not related to cancer or cancer-related syndromes, herein abbreviated as NC P/LPs (non-cancer-related pathogenic/likely pathogenic variants), in 10,389 cancer cases across multiple ancestries. We identified over twent-nine-hundred NC P/LPs and described the affected genes and population frequencies. Gene-level expression analyses revealed reduced gene expression associated with NC P/LPs. Furthermore, NC P/LPs showing reduced ASE were identified using RNA-Seq analyses and found to be enriched for nonsense variants. Splicing-assay data identified several previously classified missense variants associated with mis-splicing effects. In summary, our analysis revealed NC P/LPs in ten-thousand cancer cases and their varied expression consequences.

## Methods

### Cohort description and genetic ancestry assignment

We used the clinical data provided by TCGA PanCanAtlas and restricted analyses to those with pass-QC blood/normal sequencing data. The inclusion criteria were described by the PanCanAtlas germline working group [12], where in addition to excluding cases with PanCanAtlas blacklisted germline BAM-files, cases with less than 60% genotype concordance between sequencing variant calls and SNP-genotype data were eliminated. The final cohort consists of 10,389 cases across 33 cancer types. Then, genetic ancestry assignments for 10,353 cases were obtained from the PanCanAtlas Ancestry Informative Markers (AIM) working group [13]. Of the remaining 36 cases, 10 cases were further classified based on the principal component analysis (PCA) in the TCGA PanCanAtlas Germline project [12], and the other 26 cases without genetic ancestry assignments in both projects were considered as the “other” group.

Principal component (PC) values for each case were obtained from the TCGA PanCanAtlas Germline project [12]. Briefly, the downloaded PC data were calculated based on 298,004 variants with MAF > 0.15 and low missingness. The PC1 and PC2 accounted for 51.6% and 29.2% of the variations across the first 20 PCs, and none of the trailing PCs accounted for more than 3.2%. Thus, we subsequently controlled for PC1 and PC2 in gene expression analysis.

### Variant identification and classification

We downloaded CharGer-prioritized variants among the ~ 1.46 billion germline variant calls conducted by TCGA PanCanAtlas Germline working group [12] and further required the variants to have sufficient DNA variant read counts (≥ 5 alternative allele read counts and ≥ 0.2 alternative allele read frequency in both the normal and tumor bam file) retaining 35,911 prioritized germline variants (sample-variant). We then filtered out variants associated with cancer or cancer syndromes, retaining 23,928 sample-variants. Non-cancer-related variants were considered as those not in the curated 152 cancer predisposition genes by the PanCanAtlas germline group (Additional file 1: Table S1) nor have “tumor,” “cancer,” “neoplasia,” or cancer-related “-oma” terms in their corresponding ClinVar trait. We noted that the systematic assessment of the 23,928 variants may miss those showing “Conflicting interpretations of pathogenicity” or with updated classification on ClinVar [14] since the PanCanAtlas study [12] and/or those showing little other supporting evidence in the ACMG guideline [1]. Some of these variants could be P/LPs upon close examination and, particularly, the variants in the ACMG 59 genes could have critical clinical implication. Thus, we further extracted all variants in the 34 non-cancer ACMG 59 genes from the original variant call data, filtered with ≥ 5 alternative allele read counts, and conducted variant interpretation.

Variant interpretation of all the unique non-cancer variants was assigned using the standard ACMG classification criteria [1] and variant reviews. Variant interpretation was first screened using our in-house bioinformatics pipeline based on InterVar [15]. The pipeline automatically collected 18 out of 28 lines of evidence used in the ACMG guidelines [1]. Potential P/LP variants identified by the pipeline were then manually reviewed by a board-certified molecular geneticist using the ACMG classification criteria. A total of 2920 counts were identified in 757 unique P/LP variants in 363 NC genes. The majority (96.0%) of these variants were reported in ClinVar [14] as P/LP. For the remaining newly identified P/LP variants, 19 (63.3%) of them are rare predicted loss-of-function variants. Eleven newly identified missense P/LP variants were classified based upon functional studies provided in the literature, protein domain, rareness in general populations, P/LP variants at the same residue, and computational predictions.

Altogether, the procedure resulted in 2271 unique (2920 sample-variants) NC P/LPs (Additional file 2: Table S2). Using Online Mendelian Inheritance in Man (OMIM), we annotated associated disease and mode of inheritance to genes with ACMG-classified NC P/LPs.

### gnomAD analysis

We analyzed the variant-level frequency of our identified NC P/LPs using variants of 118,479 non-cancer exomes sample that pass all filters in the genome aggregation database (gnomAD-non-cancer) v.2.1 [16].

### Gene expression impact analysis

We downloaded a batch-corrected and normalized TCGA expression data processed by TCGA PanCanAtlas from Genome Data Commons (GDC). We calculated tumor expression percentile of individual genes in each cancer cohort by using the empirical cumulative distribution function (ecdf), as implemented in R, and downloaded consensus measurement of tumor purity information by using TCGAbiolinks R package. Next, we used a multivariate linear regression model to assess the impact of NC P/LPs on expression of affected genes with at least three carriers in the pan-cancer cohort, where the changes in gene *i* mRNA expression, *y*_*i*_, were a linear function of variant status and covariates (including age, gender, tumor purity, PC1, PC2, and cancer type) *x*_*i*_:

$$ {y}_i={\beta}_0+{\beta}_i{x}_i+{\varepsilon}_i,i=1,\dots, n. $$

where *β*_0_ was the intercept, and *β*_*i*_ was the coefficient for variant status and covariates. Samples lacking the required covariates or gene expression for the multivariate linear model are not considered. A total of 7734 cases and 2005 NC P/LPs had complete data for the model. Benjamini-Hochberg (BH) procedure was performed to adjust *P*-value into FDR (false discovery rate). We selected 0.05 and 0.15 as the FDR cutoff for significant or suggestive associations affected by NC P/LP, respectively.

### Allelic specific expression (ASE) analysis

Barcodes of TCGA patients were mapped to file ids for RNA-seq BAM files. To circumvent a potentially large multiple-testing correction penalities, we constrained the analyses to rare (MAF ≤ 0.05%) NC P/LPs. For 2640 patients with cancer variants or NC P/LPs, 3625 file ids for RNA-seq BAMs were mapped. For 118 TCGA patients, RNA-seq BAM files were not found. GRCh37 aligned BAM files were downloaded from TCGA Legacy Archive using the gdc client and bam-readcount v0.8.0 was used to extract read counts for GRCh37 variants ids for each specified patient by providing variant id location and BAM file. Read counts for each base (A,G,C,T) were compiled across all BAM files. When multiple RNA-Seq Bam-files are available for one case, we selected the Bam-file with the deepest sequencing depth at that allele for further analysis. We retained variants with at least six read counts (reference allele + alternate allele ≥ 6) for ASE analysis. Next, we conducted a one-sided binomial test with a null probability of success 0.5 in a Bernoulli experiment to identify ASE, where the alternative allele shows significantly less expression. Subsequently, we used BH to adjust *p*-value into FDR and defined FDR less than 0.05 and 0.15 as significant and suggestive, respectively.

Furthermore, the gene-level enrichment of variants with significant ASE is assessed by constructing a two-by-two table of variants located in a gene region versus ASE status and conducting a two-sided Fisher exact test. We limited the analysis to genes with at least 3 significant ASE NC P/LPs and more than 70% of carriers showing significant ASE. *P* value is adjusted by BH into FDR and two FDR cutoffs, 0.05 and 0.15, are selected to define significant and suggestive enrichment, respectively.

We used an empirical permutation-based method to evaluate the enrichment of NC P/LPs for each predicted variant function class, where the test statistics is the percentage of significant ASE NC P/LPs. The ASE statuses were randomly shuffled for 10,000 times, and each time, the percentage of significant ASE NC P/LPs was calculated. We defined *P* value for each predicted variant function class as the fraction of significant ASE NC P/LP proportions under random conditions that were greater than the observed one.

### Variants showing mis-splicing effects

We utilized the data from our recently published Massively Parallel Splicing Assay (MaPSy) experiment [17]. Based on the raw allelic counts of the reference and induced alternate allele, we calculated the allelic ratio and significance for each candidate splicing variant. Variants with BH-adjusted FDR less than 0.05 and allelic ratio less than -log2(1.5) in both the in vitro and in vivo assays were regarded as variants showing mis-splicing effects. We then match these variants based on their genomic coordinate, reference, and alternative alleles to variants we identified in TCGA patients.

## Results

### NC P/LPs in 10,389 cancer cases

**Fig. 1.**
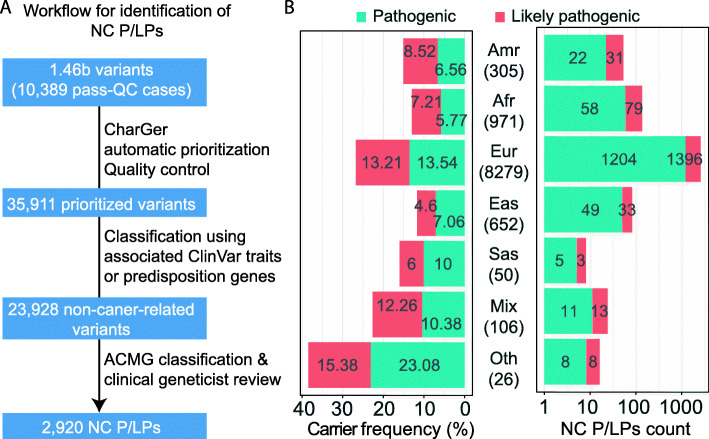
Non-cancer-related pathogenic/likely pathogenic variants (NC P/LPs) in over ten thousand cancer cases. **A** Schematic overview for identification of NC P/LPs in 10,389 cancer cases. **B** Frequency of NC P/LP carriers (left panel) and count of NC P/LPs (right panel) across different ancestries. The total case number of each ancestry is labeled

We identified NC P/LPs in the TCGA cohort of 10,389 adult cancer cases across different ancestral populations. The cohort contained germline genome data that passed quality control procedures as described by TCGA PanCanAtlas Germline working group [12]. Genetic ancestry analyses of PanCanAltas AIM and Germline working groups [12, 13, 18] stratified the cohort into 305 individuals of the Latinx/Native American, 971 of the African American, 8279 of the European, 652 of the East Asian, 50 of the South Asian, 106 of mixed (> 20% genetic admixture) ancestries, and 26 of other ancestry (Methods, Fig. 1).

In the 10,389 cases, TCGA PanCanAtlas Germline working group previously conducted variant calling that resulted in ~ 1.46 billion variants and utilized CharGer [19] to prioritize variants [12]. Among the CharGer-prioritized variants as well as all the variants in the ACMG 59 genes that are not directly associated with cancer, we systematically filtered them based on allele read count thresholds and association with cancer or cancer syndromes, followed by ACMG classification along with variant reviews by a board-certified molecular geneticist (Methods). Based on this procedure, we identified 2916 heterozygous and 4 homozygous NC P/LPs that were pathogenic or likely pathogenic (Fig. 1A). These NC P/LPs included 753 unique variants distributed across 363 non-cancer genes, harbored in 24.1% (2505/10,389) of the cancer cases (Additional file 3: Table S3). We further examined the frequencies across ancestries: 26.75% of European ancestry were found to harbor NC P/LPs, a higher frequency compared to other ancestries with more than 100 patients, including 15.08% of the Latinx/Native American, 12.98% of the African American, and 11.66% of the East Asian (Fig. 1B). Overall, putative autosomal dominant (AD) variants affected 2.07% (215/10,389) and autosomal recessive (AR) variants affected 22.02% (2288/10,389) of the cohort while showing different frequencies across ancestries (Additional file 4: Figure S1).

Among the ACMG 59 genes [2], for which reporting of secondary findings is recommended, 34 were not directly cancer-related. These 34 ACMG non-cancer genes affected 1.48% of the TCGA cancer cases across all ancestral groups. Restricting to the P/LPs in genes that are not directly cancer-related, previous studies have reported 1.0% (95 % confidence interval (CI) 0.4–1.6%) carriers in 1000 Genome project [20], 1.2% (1.0–1.3%) in eMERGE [21], 1.1% (0.1–2.0%) in European American Framingham Heart Study, and 0.5% (0.2–0.7%) in African-American Jackson Heart Study [22]. The carrier frequencies may vary depending on genes assessed, evidence available at the time of pathogenicity assignment, ancestry of participants, and participant ascertainment method.

Using 94 AR disorders assessed by Haque et al. [23], we found TCGA carrier frequencies of 10.1% (95 % confidence interval (CI) 9.5–10.8%), 6.2% (CI 4.7–7.7%), 1.7% (0.7–2.7%), and 2.6% (0.8–4.4%) in individuals of European, African American, East Asian, and Latinx ancestry for at least one P/LP variant in the 94 disorders. Our estimates are slightly higher in European-ancestry individuals and lower in African American, East Asian, and Latinx compared with Haque et al. [23] reported carrier frequencies of 7.7% (7.5–7.9%), 11.3% (10.9–11.7%), 6.9% (6.3–7.6%), and 6.0% (5.6–6.4%), in the same ancestral groups. The differences between the two studies in part reflect differences in knowledge and standards for classification of variants over time.

### Prevalence of NC P/LPs across ancestral populations

**Fig. 2:**
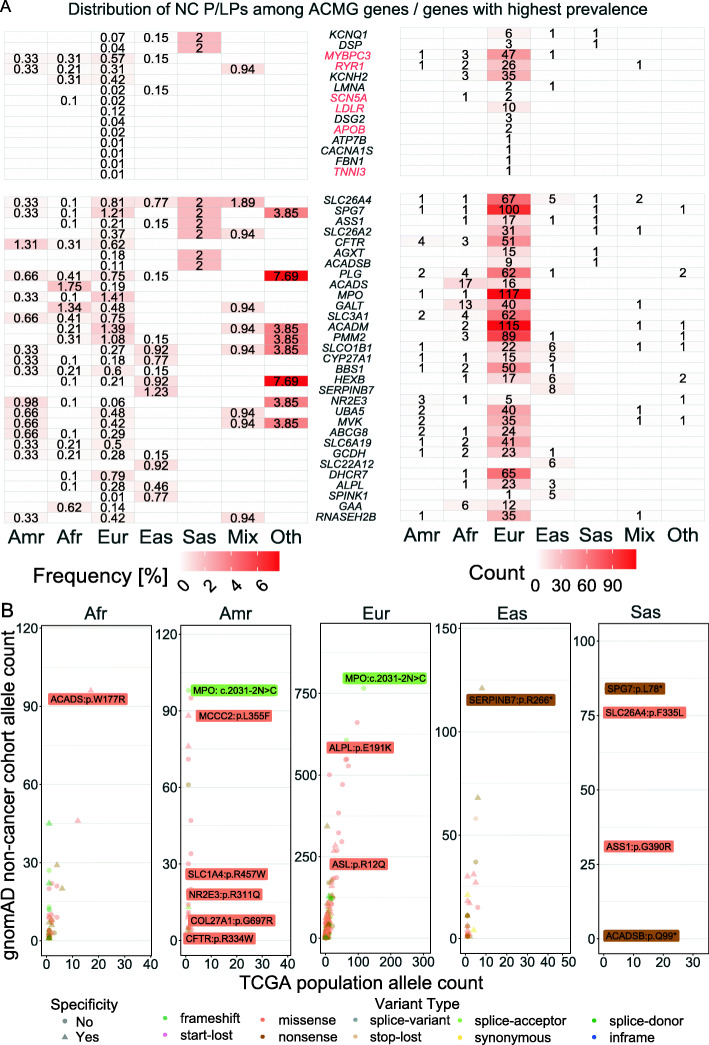
Prevalence of affected genes across ancestries. **A** Frequency/count of NC P/LP carriers in each ancestry among the ACMG 59 genes and the top 10% genes (ranked by sums of all defined ancestry frequencies, excluding Mix and Other). The ACMG genes affected were shown at the upper panel while the top 10% genes at the lower panel. The genes associated with autosomal-dominantly inherited diseases were labeled red. **B** Variant count of predisposing variants in the matched gnomAD ancestry (European of gnomAD is the union of FIN and NFE populations). TCGA population-specific NC P/LPs, exclusively found in a specific TCGA ancestry, are shown as a triangle. Top NC P/LP or top TCGA ancestry-specific NC P/LP, ranked by allele counts in TCGA or gnomAD, was labeled

We investigated the genes with the highest prevalence of NC P/LPs across ancestries. Fourteen of the ACMG 59 genes were affected by NC P/LPs in TCGA (Fig. 2A). For example, NC P/LPs in *KCNQ1*, associated with type 1 long QT syndrome, affected 6 European individuals and 1 individual in East Asian or South Asian in the TCGA cohort. Many ACMG 59 genes were restricted to individuals of European population in this cohort, such as *LDLR*, *DSG2*, *APOB*, *ATP7B*, *CACNA1S*, *FBN1*, and *TNNI3* (Fig. 2A and Additional file 5: Table S4).

Across populations, the most commonly identified predisposing genes—each with at least 100 carriers—included *SPG7*, *MPO*, and *ACADM*; all three genes are associated with autosomal recessively inherited diseases (Fig. 2A and Additional file 5: Table S4). *SPG7* variants were observed in 100 European carriers (1.21%) and one carrier each of Latinx/Native American, African American, and South Asian ancestry. Similar to *SPG7*, *MPO* variants were found in 117 European carriers (1.41%), one carrier of Latinx/Native American and African American ancestry. *ACADM* variants were identified in 115 European carriers (1.39%), 2 carriers of African American and one carrier of mixed ancestry. We also observed several commonly ancestry-specific genes carrying variants that showed occurrences exclusively in one ancestral population. In addition to those exclusively identified in the Europeans, we found two ACMG 59 genes whose NC P/LPs only affected East Asians in TCGA, including *SERPINB7* in 8 carriers (1.23%) and *SLC22A12* in 6 carriers (0.92%) (Fig. 2A and Additional file 5: Table S4). These results highlight the genetic variants that may disproportionally affect diverse populations.

We sought validation of NC P/LPs in the gnomAD non-cancer cohort (abbreviated as gnomAD below), which had no samples overlapping with the TCGA cohort. The carrier frequencies of the TCGA-identified NC P/LPs were the highest among the gnomAD Ashkenazi Jewish (ASJ), Non-Finnish European (NFE), and Finnish (FIN) compared to other ancestral populations (Additional file 4: Figure S2), consistent with ClinGen’s recent report finding Europeans in gnomAD contained over half of the information on the clinically relevant variants on ClinVar [24]. We also examined the gene-level carrier frequencies of the same variants across different gnomAD ancestral populations. Multiple genes, including *SPG7*, *MPO*, and *ACADM,* showed higher carrier frequencies of this set of NC P/LPs in European individuals from both the TCGA and gnomAD cohorts (Fig. 2A and Additional file 4: Figure S3A). *ACADS* and *GALT* variants were present at highest frequency in African Americans in both TCGA and gnomAD; similarly *SERPINB7* and *SLC22A12* variants appeared predominantly in the East Asian population of both cohorts.

We further investigated the concordance of variant-level frequencies in the matched ancestries between TCGA and gnomAD. We found significant correlations of variant frequencies in East Asian (Pearson *R* = 0.8, *p* value = 2.53e−07), European (Pearson *R* = 0.83, *p* value = 4.13e−143), and African American (Pearson *R* = 0.82, *p* value = 1.35e−12) population, whereas the variant frequencies in Latinx/Native American ancestry did not show significant correlations (Pearson *R* = 0.09, *p* value = 0.58, Additional file 5: Table S4), likely due to the smaller sample size or admixtures in the populations. Multiple variants found exclusively in one TCGA ancestry were rediscovered in their respective ancestry cohort of gnomAD, such as *ACADS* p.W177R in African American, *MCCC2* p.L355F in Latinx/Native American, *ALPL* p.E191K in European, *SERPINB7* p.R266* in East Asian, and *ACADSB* p.Q99* in South Asian (Fig. 2B and Additional file 5: Table S4). Replicated across cohorts, the markedly higher frequencies of these variants in one population compared to the others support their population specificity and potential founder effects.

We further compared the frequency of each NC P/LP in TCGA vs. gnomAD stratified by population using a two-tailed Fisher’s exact text. The analyses identified 57 variants (FDR < 0.05) (Additional file 6: Table S5), the majority of which were TCGA-enriched variants found in the European ancestry and the most significant ones included *MPO* c.2031-2A>C (splice site variant, myeloperoxidase deficiency), F11 p.E135* and p.F301L (hereditary factor XI deficiency disease), and *ACADM* p.K333E and p.G271R (medium-chain acyl-coenzyme A dehydrogenase deficiency). In contrast, *CYP21A2* p.P454S, *TSFM* p.Q307* and ALPL p.E191K showed higher frequencies in gnomAD compared to TCGA Europeans (Additional file 4: Figure S3B). Acknowledging the caveat of comparing TCGA vs. gnomAD data from different sequencing platforms, variant calling pipelines, and sampled sources and populations, these results suggest a potential different distribution of NC P/LPs that may be indirectly associated with the cancer phenotype that needs to be further tested.

### Gene expression impacted by NC P/LPs

**Fig. 3:**
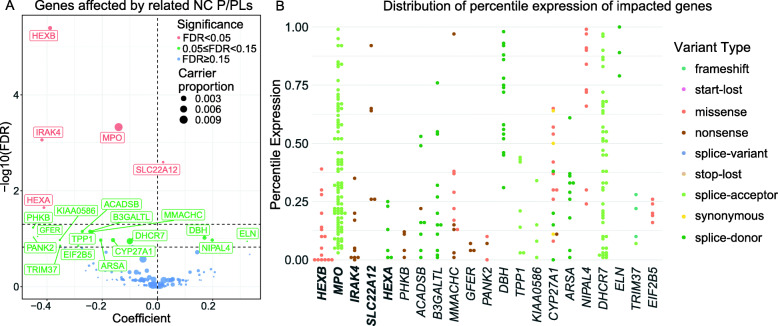
Impact of NC P/LPs on gene expression. **A** A volcano plot showing genes whose expression is affected by related NC P/LPs. Color represents the significance level, and the size represents the gene’s NC P/LP carrier frequency. **B** Distribution of percentile expression in a specific cancer at NC P/LP carriers of genes whose expression is significantly/suggestively impacted by NC P/LPs. Color represents variant type. Significant genes is highlighted in bold

Many of the identified NC P/LPs are truncating variants that are presumed to alter expression of the gene products through mechanisms such as NMD. To interrogate the variant consequences at the gene expression level, we applied a multivariate linear regression model using the expression quantile of the affected gene within each cancer cohort as a dependent variable and variant status as the independent variable, adjusting for age, gender, PC1, PC2, cancer type, and tumor purity (Methods). We found 5 significant and 16 suggestive genes to be differentially expressed, 17 of which showed reduced expression in variant carriers (Fig. 3A).

We further examined the specific variants that co-occurred with low gene expression in the bottom quartile of their respective cancer cohorts (Fig. 3B and Additional file 4: Figure S4). The majority or all nonsense variants carriers of multiple genes showed bottom-quartile expression of the affected genes, such as *IRAK4*, *PHKB*, *MMACHC*, and *GFER*. Meanwhile, bottom-quartile *HEXB* gene expression was observed in 13 carriers of the missense variant *HEXB* p.P417L. Overall, we observed distinct expression effects of different variant types. Among 376 nonsense variants, 137 (36.4%) showed bottom-quartile expression of the respective genes, confirming the potential effects from NMD. In comparison, 271 of 1169 (23.2%) missense variants also showed bottom quartile expression (approximating the 25% in a null scenario, Additional file 7: Table S6), suggesting that many of these missense variants, along with a fraction of truncating variants, may exert their effects through mechanisms independent of altering gene expression.

### Allele-specific expression of NC P/LPs

**Fig. 4:**
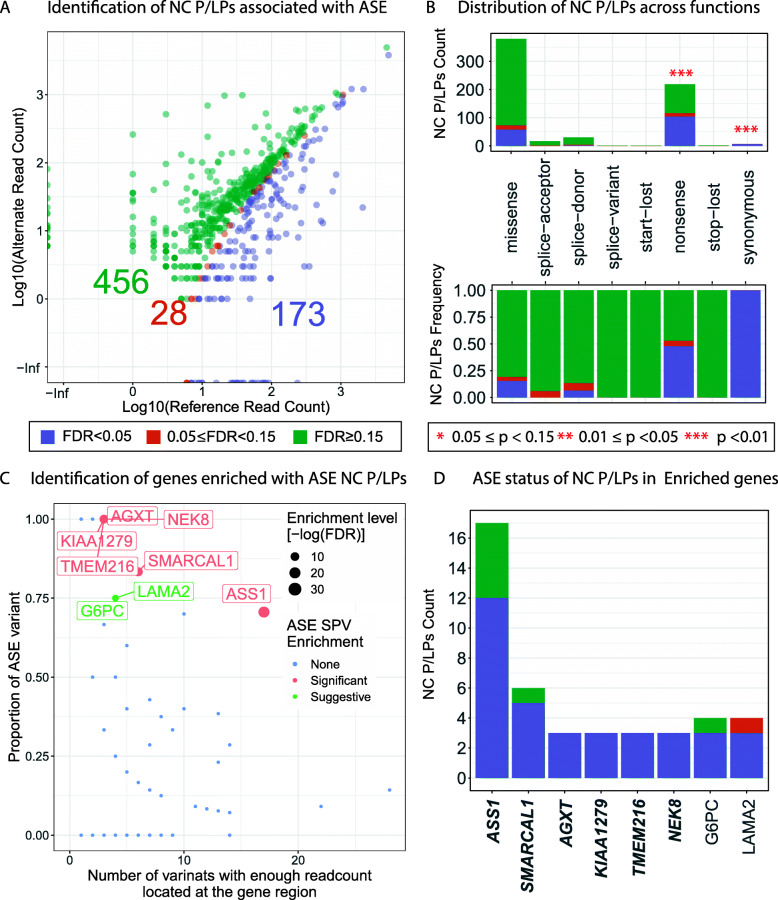
Rare variants showing allele-specific expression (ASE) and enriched genes. **A** Identification of rare NC P/LPs associated with ASE. Color represents ASE status. **B** Distribution of NC P/LPs with distinct ASE enrichment status across predicted variant function classes. **C** Gene enrichment analysis for NC P/LPs showing significant ASE. Each dot is a gene affected by NC P/LPs. The *X*-axis represents the number of NC P/LPs with sufficient read counts located at a specific gene region, of which significant ASE NC P/LP proportion is *Y*-axis. **D** Count of NC P/LPs with distinct ASE enrichment status for ASE NC P/LP enriched genes. Significant genes is labeled bold. Color of **B** and **D** were the same as **A**

The majority of differentially expressed genes associated with NC P/LPs showed reduced expression (Fig. 3A). Further, many pathogenic variants are assumed to alter gene expression through mechanisms such as NMD, leading that ASE at the RNA-level would be observed for a heterozygous individual. To further assess the mechanism at a variant level, we further identified ASE showing reduced expression of the alternate allele for the rare NC P/LPs (MAF ≤ 0.05%) by performing a one-sided binomial test (Methods). Among the 657 rare NC P/LPs with sufficient read counts in tumor RNA-Seq data for analyses, ~ 26.3% (173/657) showed significant ASE (FDR < 0.05), and another 4.26% (28/657) showed suggestive ASE (0.05 ≤ FDR < 0.15) (Fig. 4A).

We investigated whether the ASE status of variants were disproportionally present in a predicted variant function class by conducting a permutation test (Methods). Among the predicted variant function class, nonsense variants were significantly enriched for those showing ASE (48 %, *P* < 1E−4), confirming their transcriptional impact that is likely mediated through NMD. In addition, synonymous variants are also enriched for those showing significant ASE (7 out of 7, *P* < 1E−4), although there were only 7 synonymous variants in our analysis (Fig. 4B and Additional file 8: Table S7), including four East Asian carriers of *CYP27A1* c.862G>T, one East Asian carrier of *G6PC* c.727G>T and two European carriers of *DGUOK* c.676G>A.

**Fig. 5:**
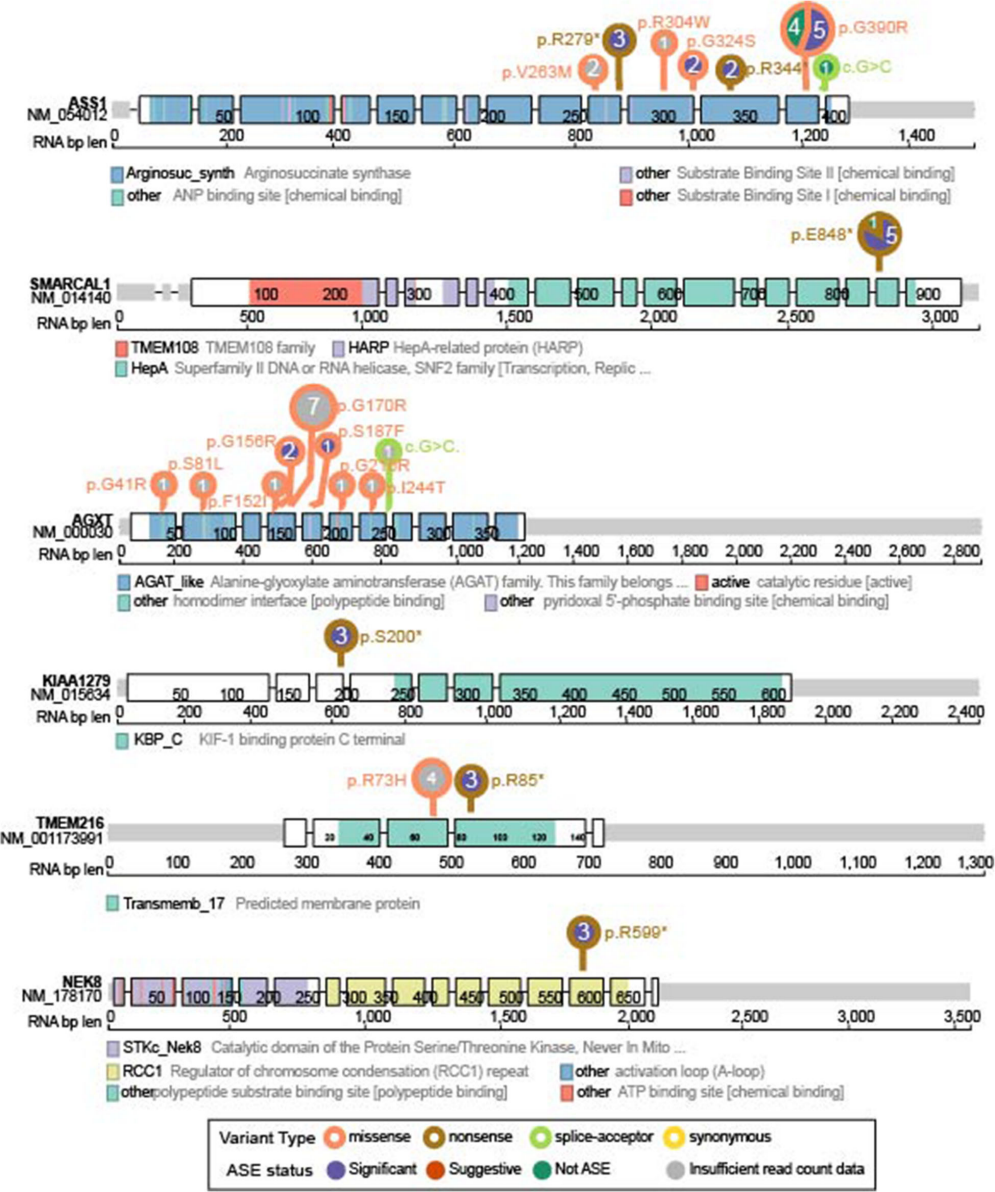
Lolliplots showing the positions of NC P/LPs in genes significantly enriched with significant ASE NC P/LPs. NC P/LPs were labeled by the HGVSp symbol. Different colors on the gene product bar depict different protein domains. The number above variant loci shows the number of carriers with distinct ASE enrichment status (indicated by fill color). The stroke colors of the circle indicates predicted variant function classes

Next, we identified genes enriched for significant ASE NC P/LPs using Fisher’s exact test (Methods). We observed that ASE variants were significantly enriched in 6 genes, including *ASS1* (FDR = 0.001), *SMARCAL1* (FDR = 0.022), *AGXT* (FDR = 0.023), *KIAA1279* (FDR = 0.023), *TMEM216* (FDR = 0.023), and *NEK8* (FDR = 0.023) (Fig. 4C). At the variant level, 12 of the 17 *ASS1* carriers showed significant ASE, including two carriers of missense variant p.G324S, nonsense variant p.R344*, all three carriers of the nonsense variant p.R279* and five carriers of missense variant p.G390R (Figs. 4D and 5). Five out of the six *SMARCAL1* p.E848* carriers also showed significant ASE. All carriers with enough read counts data of the remaining four genes (*AGXT*, *KIAA1279*, *TMEM216*, and *NEK8*) showed significant ASE (Figs. 4D and 5). Additionally, we identified two genes showing suggestive enrichment of significant ASE NC P/LPs, including *G6PC* and *LAMA2* (FDR = 0.057) (Fig. 4C). For *G6PC*, significant ASE was found in one each carrier of p.R83C, p.L216, and p.Q347* (Fig. 4D and Additional file 4: Figure S5). For *LAMA2*, all 3 carriers of p.R1326* showed significant ASE, while the only carrier of p.R1826* showed suggestive ASE (Fig. 4D and Additional file 4: Figure S5). There is no overlap between genes enriched with ASE variants and genes whose expression is associated with variants. The non-concordance between ASE analysis and gene expression impact suggested a possible expression compensation from the reference allele or a lack of power in the gene expression analysis. Altogether, the observed ASE validated the expression consequences of many NC P/LPs.

### Mis-splicing effects induced by NC P/LPs

Aside from nonsense variants assumed to undergo NMD, we also observed significant numbers of variants showing ASE and gene expression effects (Figs. 3 and 4). Recent studies have shown that, in addition to canonical splice sites, many missense variants can also affect splicing [17, 25]. Utilizing data from our recently published Massively Parallel Splicing Assay (MaPSy) experiment surveying nearly five thousand variants [17], we systematically identified variants showing mis-splicing effects both in vitro and in vivo matched to the NC P/LPs identified in TCGA patients. Among 226 NC P/LPs that overlapped with MaPSy variants, 36 NC P/LPs showed significant mis-splicing effects, including 30 European carriers of missense *GALT* p.Q103R, one European carrier of missense *SMPD1* p.H423Y, one American carrier of missense RYR1, two European carriers of nonsense *NDUFAF2* p.R47*, one European carrier of nonsense *HADH* p.R236*, and one East Asian carrier of nonsense *BBS1* p.E586*. The carrier of nonsense *HADH* p.R236* showing mis-splicing in MaPSy also showed significant ASE.

To discover additional variants with expression and possibly functional consequences, we assessed the mis-splicing effects that may be exerted by all prioritized variants (i.e., the 35,911 variants in Fig. 1A). Among these, 21,008 were non-cancer related NC P/LPs that yielded insufficient evidence to satisfy a likely pathogenic classification, and 266 were characterized by MaPSy. Fifteen of these prioritized missense variants showed mis-splicing effects, including three European carriers of *DARS2* p.R263Q, one European carrier of *CD36* p.I413L, one European carrier/one African carrier of *SLC29A3* p.M116R, and eight East Asian carriers/one Mixed ancestry carrier of *PLG* p.A620T. On ClinVar, *DARS2* p.R263Q is classified as pathogenic by a single submitter associated with the condition of leukoencephalopathy with brain stem and spinal cord involvement and lactate elevation, whereas *SLC29A3* p.M116R is pathogenic without assertion criteria and linked to histiocytosis-lymphadenopathy plus syndrome. The European carrier of *CD36* p.I413L, another MaPSy-identified variant associated with platelet glycoprotein IV deficiency, also showed significant ASE along with the suggestive ASE found in the European carrier of the MaPSy-identified *SLC29A3* p.M116R. These variants have additional functional evidence of mRNA effects in patient samples and/or functional assays, which strengthen variant interpretation assertions.

## Discussion

Assessment of hereditary cancer and tumor sequencing are the most common use cases for genomic medicine in adults. As genome sequencing is considered as the platform for genomic assessment, we sought to understand the expected yield of NC P/LPs variants. We present one of the largest studies to date on NC P/LPs in a cancer sequencing cohort. In the TCGA cohort of over ten thousand adult cancer cases, NC P/LPs in disease associated genes were found to be about 25% of individuals. The NC P/LPs corresponded to multiple diseases, and many genes and variants showed ancestry-specific patterns validated across the TCGA and gnomAD non-cancer cohorts. As expected, a higher fraction of nonsense variants showed ASEs, although variant-level ASE did not guarantee the reduced expression of the affected gene. Some missense variants were also associated with ASE or low gene expression, and a few were found to be associated with mis-splicing effects.

The frequency of variants we identified in 34 ACMG non-cancer or cancer-syndrome-related genes were slightly higher than those found in previous studies [20–23]. In addition to the intrinsic cohort difference, the differences in frequency may reflect the difference in sequencing platforms, the advances in variant interpretation over time [14, 26], and the difference in variant interpretation guidelines [27]. In particular, the TCGA dataset used herein include samples collected and sequenced over a decade ago, and the biased inclusion of larger tumors and individuals with severe cancer may not yield generalizable results for current cancer patients or the overall population.

The higher rate of NC P/LPs found in the European ancestry is consistent with previous studies and may reflect the bias of characterized and reported variants in those of European ancestry [24]. Much of the non-European populations are under-represented in existing sequencing cohorts [28–30]. Fewer counts of pathogenic or likely pathogenic variants identified in non-Europeans herein also highlight the limitation of current cohorts in identifying non-European pathogenic alleles. Current germline sequencing in non-European patients produces higher rates of variants of unknown significance (VUSs) [31, 32] and false-positives [33], limiting the clinical utility of genetic testing for those groups. Ongoing projects, including CSER [34], eMERGE III [35], UKBioBank [36, 37], TopMed [38], Million Veterans Program [39], and All of Us Research Program, are beginning to address the challenge for diverse populations. Ancestral populations in TCGA and gnomAD showed correlations in their carrier frequencies, suggesting combining evidence from these ongoing efforts can likely increase confidence in identifying critical variants within populations.

Many predisposing variants lead to aberrations of gene expression. Such expression consequences can help assess pathogenicity among VUSs, yet this evidence is under-utilized. Growing evidence suggests the genomic context, even in a local genomic region, affects eQTL associations [40–42]. Thus, as multi-omics cohorts expand to cover substantial fractions of diverse populations, evaluating expression consequences of alleles found in different ancestral groups can help facilitate the interpretation of rare variants. A caveat for the expression analyses presented herein is that most RNA-Seq data in TCGA were derived from tumor tissues, which may not be the tissue directly affected by the disease associated variant but could provide a surrogate to assess expression consequences. Additional paired samples with expression data and genomic data will help assess effects of synonymous variants on splicing and impact of regulatory variants that will improve prediction algorithms to improve variant interpretation more broadly.

Many rare variants found in disease predisposition testing panels, particularly missense variants, are still of unknown significance [43] and require alternative approaches for interpretation. Aside from canonical splice sites in introns, splicing effects were detected for a considerable fraction of *BRCA1* missense [25] and known exonic variants [17]. Systematic identification of such splicing effects using mRNA sequencing data from carriers or functional-screen experiments is critical to identify pathogenic splice variants that may be labeled as non-splice variants. In the current ACMG guidelines [1] for variant interpretation, one consideration is “the effect of a variant on gene/protein function as determined by a well-established functional assay,” adding either strong support of a pathogenic (PS3) or benign (BS3) impact. This evidence level is rarely used, and our results showed these data could be incorporated to increase the accuracy of variant classification.

## Conclusions

Our study represents one of the most extensive studies to date that evaluated NC P/LPs and their expression consequences. As clinical genomic sequencing becomes more common, particularly with the adoption of whole-exome or genome profiling technologies, the presented knowledge herein and additional studies in diverse populations are valuable to facilitate genome-medicine and accurately assess an individual’s comprehensive disease risk profile.

## Supplementary Information

**Additional file 1: Table S1.** The list of 152 cancer predisposition genes used to filter for non-cancer related variants.

**Additional file 2: Table S2.** Detailed information used in this research for the 2,271 unique variants.

**Additional file 3: Table S3.** The distribution of NC P/LPs among predisposing variants, patients and genes across different ancestries.

**Additional file 4: Figure S1.** Carrier frequency (left panel) and NC P/LPs count (right panel) of autosomal recessive (AR) and autosomal dominant (AD) genes across ancestries. **Figure S2.** Validation of identified NC P/LPs in TCGA by respective ancestral population in gnomAD. **Figure S3.** Validation of genes impacted by identified NC P/LPs in TCGA for respective ancestral population in gnomAD. **Figure S4.** Carrier density for the distribution of percentile expression of impacted genes. **Figure S5.** Lolliplots showing the positions of NC P/LPs in genes suggestively enriched with significant ASE NC P/LPs.

**Additional file 5: Table S4.** Detailed information of NC P/LPs for Fig. 2.

**Additional file 6: Table S5.** NC P/LPs with different frequency between TCGA cohort and gnomAD cohort.

**Additional file 7: Table S6.** NC P/LP carrier count/frequency of all affected genes, ASE NC P/LPs enriched genes and genes impacted by NC P/LPs across different quartile expression splits.

**Additional file 8: Table S7.** Count/Frequency of NC P/LPs among predicted function groups across different ASE enrichment status.

## Data Availability

Ancestry ascertainments for all samples in this study were obtained from the TCGA PanCanAtlas AIM analysis working group’s publication and its supplementary information files [13]. Germline variant calls used in this study are available through controlled access data release of the TCGA PanCanAtlas germline working group [12] (https://gdc.cancer.gov/about-data/publications/PanCanAtlas-Germline-AWG). All additional data generated or analyzed during this study are included in this published article and its supplementary information files. All the analysis codes used by this study were deposited on GitHub [44] (https://github.com/WangZishan/TCGASecondaryPredisposition).

## Abbreviations

ACMG/AMP: American College of Molecular Genetics and Association for Molecular Pathology
AD: Autosomal dominant
AR: Autosomal recessive
AIM: Ancestry Informative Markers
ASE: Allelic specific expression
BH: Benjamini-Hochberg
CI: Confidence interval
FDR: False discovery rate
GDC: Genome Data Commons
GTEx: Genotype-Tissue Expression
MaPSy: Massively Parallel Splicing Assay
NC P/LPs: Non-cancer-related pathogenic/likely pathogenic variants
NMD: Nonsense-mediated decay
OMIM: Online Mendelian Inheritance in Man
PC: Principal component
PCA: Principal component analysis
TCGA: The Cancer Genome Atlas
VUS: Variants of unknown significance
