# Supplementary material for: Non-cancer-related pathogenic germline variants and expression consequences in ten-thousand cancer genomes
Source: Genome Med. 2021 Sep 9;13:147. doi: 10.1186/s13073-021-00964-1 (PMC8431938; doi:10.1186/s13073-021-00964-1)
Supplement: Supplementary file 4 — Additional file 4: Figure S1. Carrier frequency (left panel) and NC P/LPs count (right panel) of autosomal recessive (AR) and autosomal dominant (AD) genes across ancestries. Figure S2. Validation of identified NC P/LPs in TCGA by respective ancestral population in gnomAD. Figure S3. Validation of genes impacted by identified NC P/LPs in TCGA for respective ancestral population in gnomAD. Figure S4. Carrier density for the distribution of percentile expression of impacted genes. Figure S5. Lolliplots showing the positions of NC P/LPs in genes suggestively enriched with significant ASE NC P/LPs. [file 13073_2021_964_MOESM4_ESM.pdf]

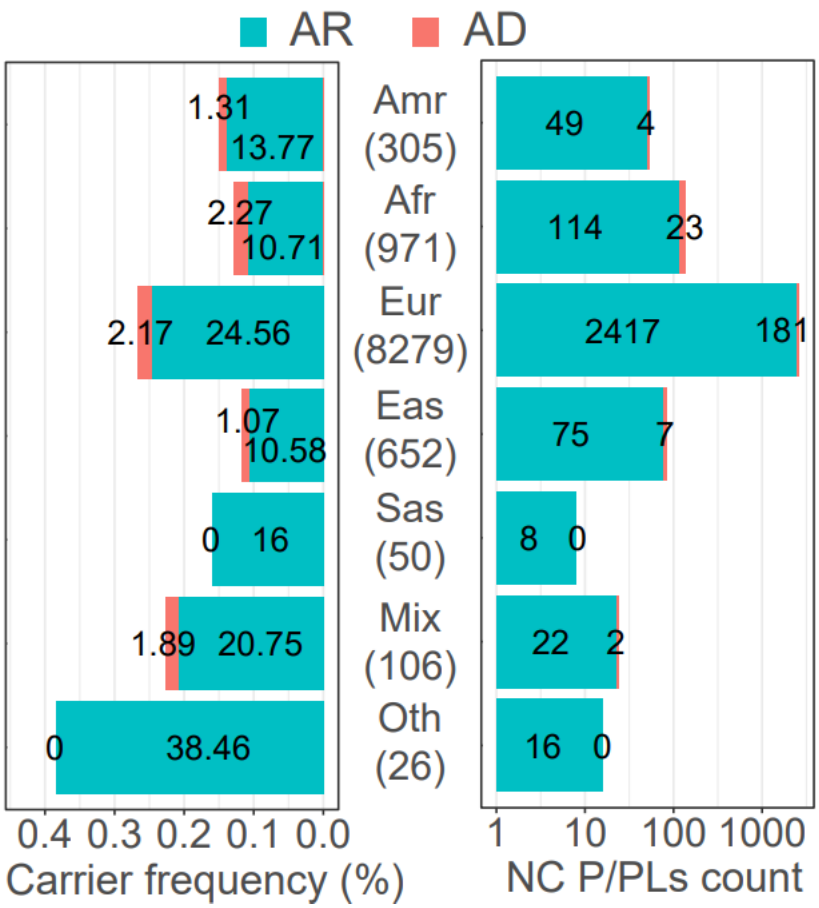

Fig. S1. Carrier frequency (left panel) and NC P/LPs count (right panel) of autosomal recessive (AR) and autosomal dominant (AD) genes across ancestries. The total case number of each ancestry is labelled.

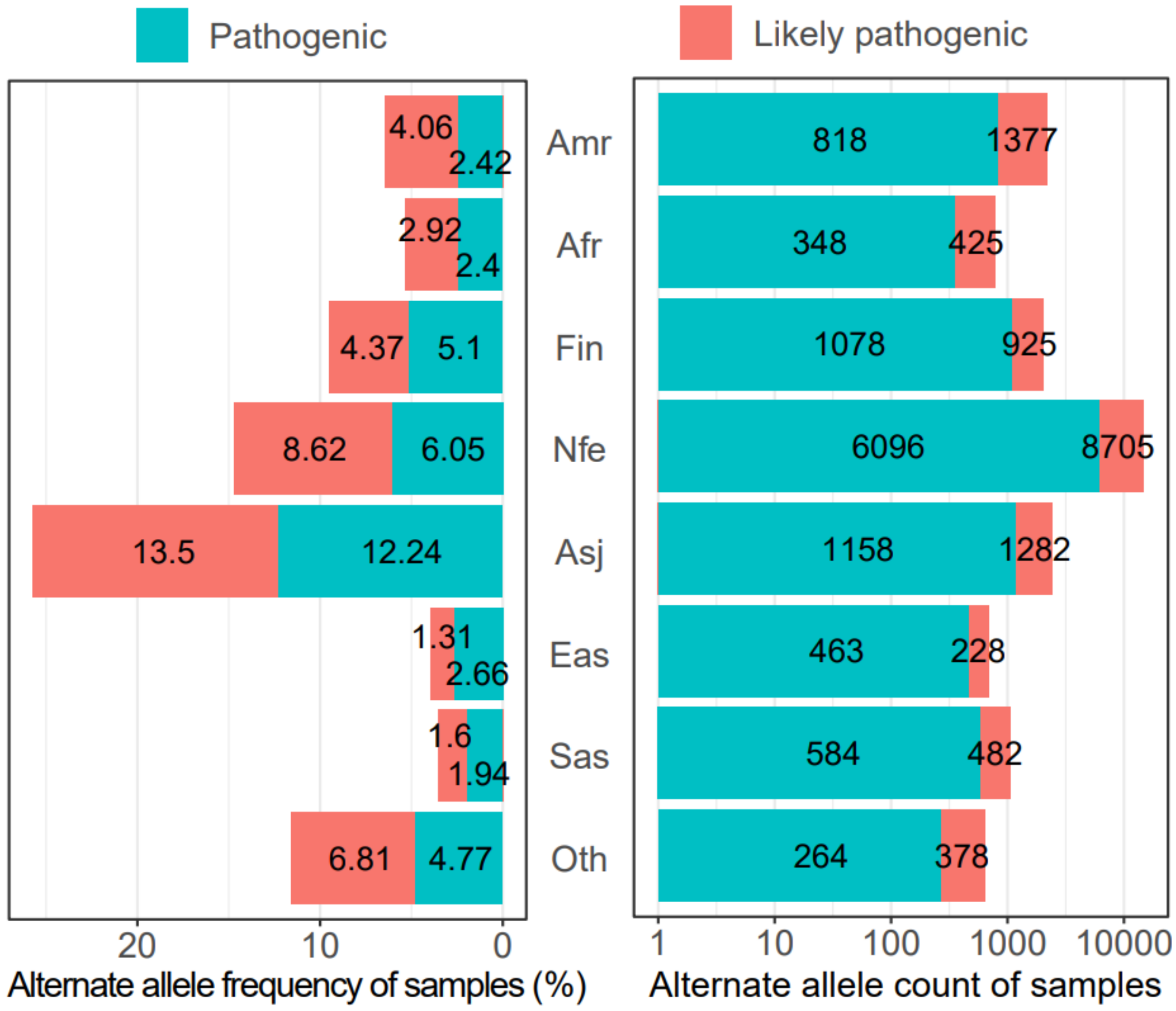

Fig. S2. Validation of identified NC P/LPs in TCGA by respective ancestral population in gnomAD. Alternate allele frequency/count of sample in gnomAD for the overlapped NC P/LPs.

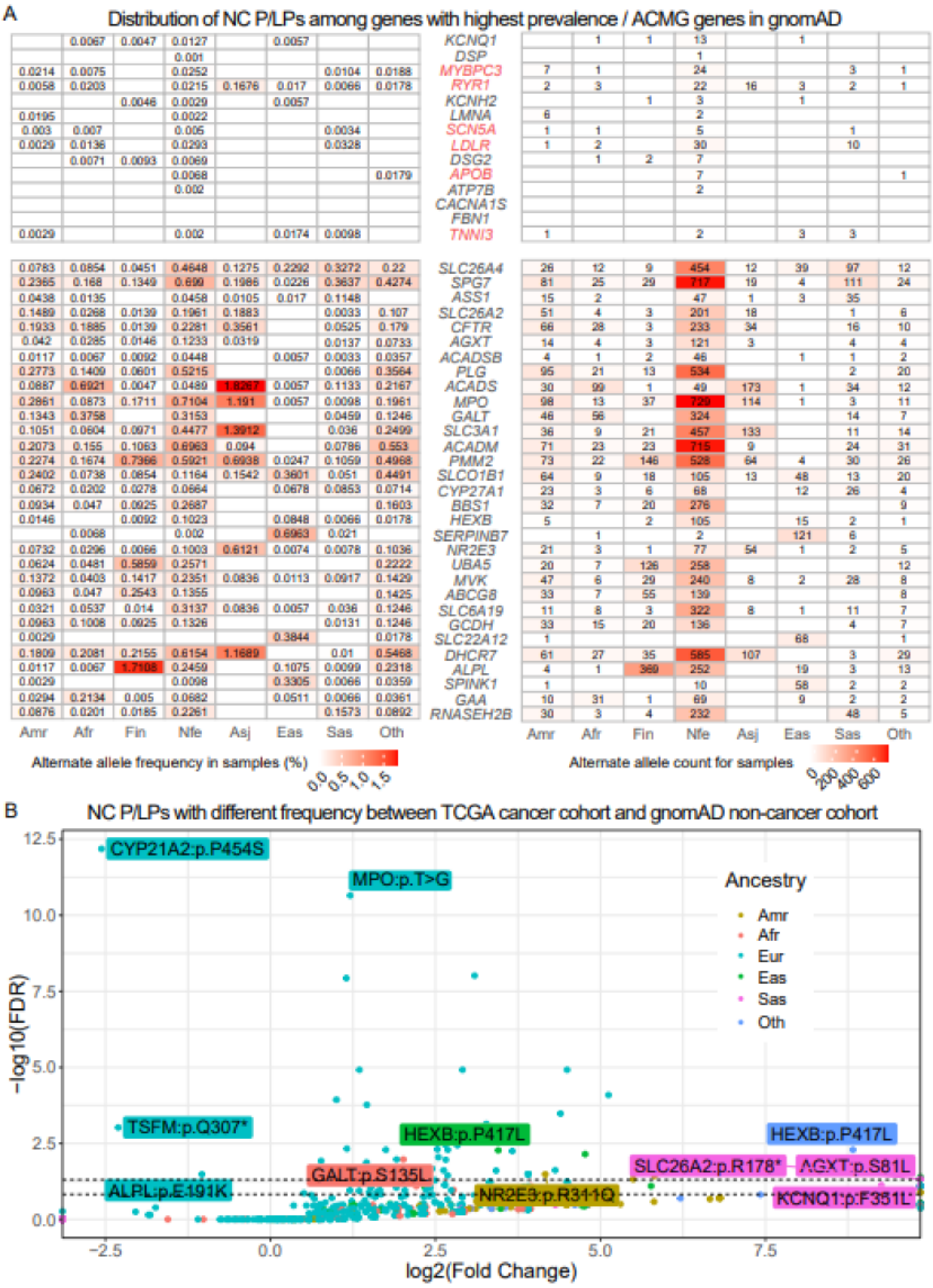

Fig. S3. Validation of genes impacted by identified NC P/LPs in TCGA for respective ancestral population in gnomAD. (A) Frequency/counts of NC P/LP carriers for predisposing genes of Fig. 2 across ancestral populations in gnomAD. (B) NC P/LPs with different frequency between TCGA cohort and gnomAD cohort. X-axis is an estimated fold change of variant frequency (frequency in TCGA/frequency in gnomAD).

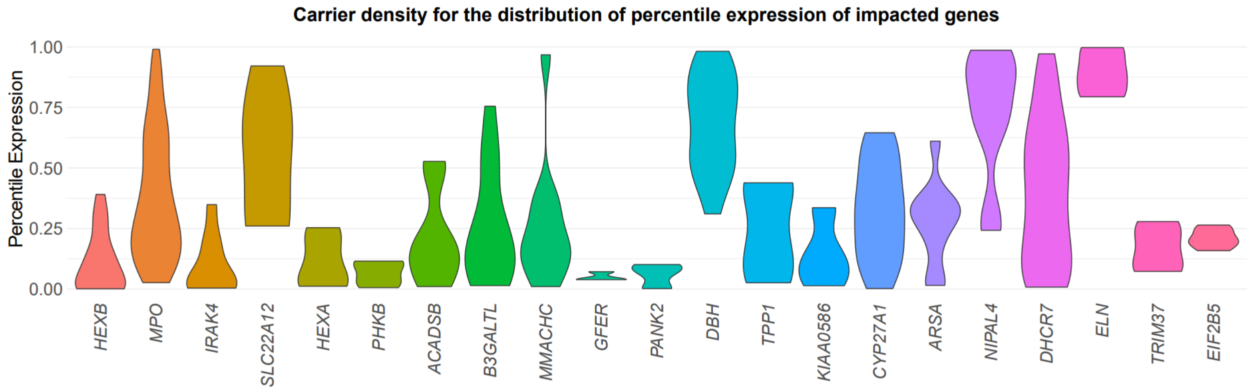

Fig. S4. Carrier density for the distribution of percentile expression of impacted genes.

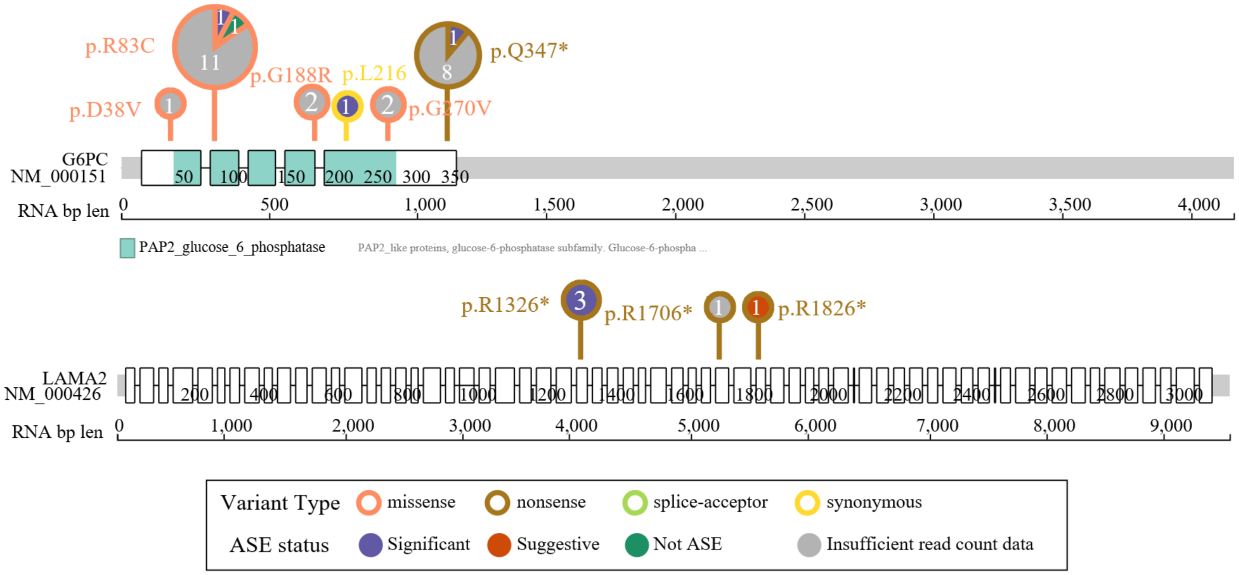

Fig. S5. Lolliplots showing the positions of NC P/LPs in genes suggestively enriched with significant ASE NC P/LPs. The meaning of symbol, color and number were the same as Fig. 5.
